# Classification of odors associated with migraine attacks: a cross-sectional study

**DOI:** 10.1038/s41598-023-35211-7

**Published:** 2023-05-25

**Authors:** Noboru Imai, Ayako Osanai, Asami Moriya, Masahito Katsuki, Eiji Kitamura

**Affiliations:** 1 Department of Neurology, Japanese Red Cross Shizuoka Hospital, 8-2 Ohtemachi, Aoi-Ku, Shizuoka, Shizuoka 420-0853 Japan; 2 Department of Neurosurgery, Itoigawa General Hospital, Itoigawa, Niigata Japan; 3 Department of Neurology, Kitasato University, Sagamihara, Kanagawa Japan

**Keywords:** Neuroscience, Neurology

## Abstract

Migraine, a common primary headache disorder, is associated with various factors such as stress, hormones in women, fasting, weather, and sleep disturbance as well as odors. We aimed to categorize odors associated with migraine and explore their relationships with clinical characteristics. A total of 101 migraineurs answered a questionnaire to determine the odors associated with migraine attacks. We performed factor analysis to explore the common factors of the odors and the relationship between these factors and the clinical characteristics. The factor analysis estimated six common factors: factor 1, fetid odor; factor 2, cooking products; factor 3, oil derivatives and others; factor 4, shampoo and conditioner; factor 5, cleaning products; factor 6, perfumes, insecticides, and rose. Factor 5 also included hair styling preparations, laundry detergent, and fabric softener, usually those with floral fragrances, and factor 5 was more likely to be associated with migraine attacks in patients with chronic migraine than in those with episodic migraine (P = 0.037). Our study showed that odors associated with migraine attacks could be categorized into six groups and suggested that some chemicals were more likely associated with migraine attacks in patients with chronic migraine than in those with episodic migraine.

## Introduction

Migraine is a common primary headache disorder with a prevalence of 8.4% in Japan^1^. Migraine attacks are believed to be provoked by various triggers such as stress, hormones in women, fasting, weather, and sleep disturbance^2,3^. Determining causality in headache triggers identified three basic assumptions, including constancy of the sufferer, constancy of the trigger effect, and constancy of the trigger presentation^4^. In clinical practice, however, these assumptions are very difficult to identify^4^. The study of agreement between self-reported triggers and early premonitory symptoms showed that some patient-reported triggers, such as light, sound, food, and skipping meals, were consistent with early premonitory symptoms^5^. This study has suggested that some patient-reported triggers may represent early brain manifestations of the premonitory phase of the migraine attack^5^. Therefore, we believe that what is considered to be a trigger is more appropriately considered an associated factor.

Odors are also common associated with migraine attacks^2,3^. Many studies have shown that odor sensitivity is a fairly specific symptom of migraine^6–12^. Although specific, odor sensitivity does not necessarily add further ability to discriminate patients in comparison to existing diagnostic criteria^13^. In contrast, few studies have reported the types of odors that are associated with migraine attacks^14–16^.

There are three anatomical systems for processing odorous stimuli, namely, the olfactory, trigeminal, and pheromone systems^15,17,18^. The pathway for each system is the olfactory nerve projection to the olfactory bulb, the trigeminal nerve projection to the somatosensory and insular cortex, and projection from the accessory olfactory bulb to the hypothalamus^15,17,18^. In addition, imaging studies showed that different odors activate different regions of the brain^19,20^.

We hypothesized that different odors are likely to cause headaches depending on age, sex, and type of migraine, such as episodic (EM) or chronic (CM). Therefore, this study aimed to categorize odors associated with migraine and to explore their relationships with clinical characteristics. We used factor analysis to find common factors in odors associated with migraine and cluster analysis to explore the relationship between odors and migraine onset/severity.

## Results

### Participants

One hundred two patients were enrolled in this study. However, one patient was excluded because of insufficiently described data. Table 1 presents the demographics of the 101 patients who participated; their ages ranged from 22 to 75 years. Of the 16 patients (15.8%) with CM, 12 (75.0%) had concomitant medication overuse headache. Odors were associated with migraine attacks in 79 (78.2%) patients. There were no significant differences in age, sex, or types of migraine with or without the onset of migraine attacks by odors.

**Table 1:** Demographics of the participants.

	All (n = 101)	Migraine attacks associated with odors	
		Yes (n = 79, 78.2%)	No (n = 22, 21.8%)
Age (years)	46.2 ± 10.9	46.2 ± 10.8	46.2 ± 11.9
Sex			
Female	90 (88.1)	71 (89.9)	19 (86.4)
Male	11 (10.9)	8 (1.01)	3 (13.6)
Migraine type			
EM	85 (84.2)	66 (83.5)	19 (86.4)
CM	16 (15.8)	13 (16.5)	3 (13.6)

Data are presented as mean ± standard deviation or n (%).

*EM* episodic migraine, *CM* chronic migraine.

### Details of odors reported to be associated with migraine attacks

The most frequently reported odors associated with migraine attacks were perfume (55.4%), tobacco (47.5%), fabric softener (32.7%), body odor (32.7%), garbage (24.8%), hairdressing products (22.8%), cars (22.8%), and sweat (19.8%) (Table 2). No patient reported migraine attacks associated with the scent of lemon, tangerine, or apple. The most frequent odors aside from the 35 items of the questionnaire were ikebana (Japanese flower arrangement) (5.9%) and gasoline (3.0%).

**Table 2:** Details of odors or odors reported to be associated with migraine attacks.

Odors	Rate of association (%)
Perfumes	56.4
Tobacco	47.5
Fabric softener	32.7
Body odor	32.7
Garbage	24.8
Hairdressing products	22.8
Automobiles	22.8
Sweat	19.8
Garlic	16.8
Rice	15.8
Grilled fish	15.8
Alcohol	14.9
Excrement	14.9
Machine oil	13.9
Vomit	13.9
Chemicals	11.9
Propane gas	10.9
Animals	10.9
Coffee	9.9
Laundry detergent	8.9
Grilled meat	8.9
Shampoo	7.9
Roses	7.9
Insect repellent	7.9
Rinse	6.9
Mint	5.9
Socks	5.0
Soap	4.0
Curry	4.0
Cheese	4.0
Hinoki	4.0
Black tea	1.0
Lemon	0.0
Tangerine	0.0
Apple	0.0

The most commonly reported places associated with migraine attacks were offices (55.4%), followed by homes (40.6%), restaurants (28.7%), and hospital waiting rooms (5.0%). No patient reported migraine attacks in the consultation or examination room. The most frequent places besides the questionnaire were on the train (7.9%), in town (5.9%), and in department stores (5.0%).

Forty-two percent of the patients used masks, 22.8% used air cleaner, and 21.8% used deodorant spray to prevent odors. In the free-text field, 16.8% of the patients reported moving out of the place, whereas 5.0% reported holding their noses with a handkerchief.

The relationships between odors and age, sex, or types of migraine are described below. Two items showed significant age differences with or without odors. The patients with migraine attacks associated with tobacco or soap were significantly younger than those without (43.0 ± 10.3 vs. 49.0 ± 10.8, P = 0.005; 34.0 ± 8.2 vs. 46.7 ± 10.7, P = 0.022). Only women had migraine attacks associated with body odor (37%) and garbage (28%) (P = 0.014 and P = 0.004, respectively). Fabric softener, sweat, socks, coffee, excrement, vomit, and animals were associated with migraine attacks in significantly more patients with CM than those with EM (55.4% vs. 27.7%, P = 0.028; 43.6% vs. 14.9%, P = 0.009; 24.8% vs. 6.9%, P = 0.028; 49.5% vs. 7.9%, P < 0.001; 30.7% vs. 10.9%, P = 0.028; 30.7% vs. 6.9%, P = 0.004).

### Factor analysis

**Figure 1:**
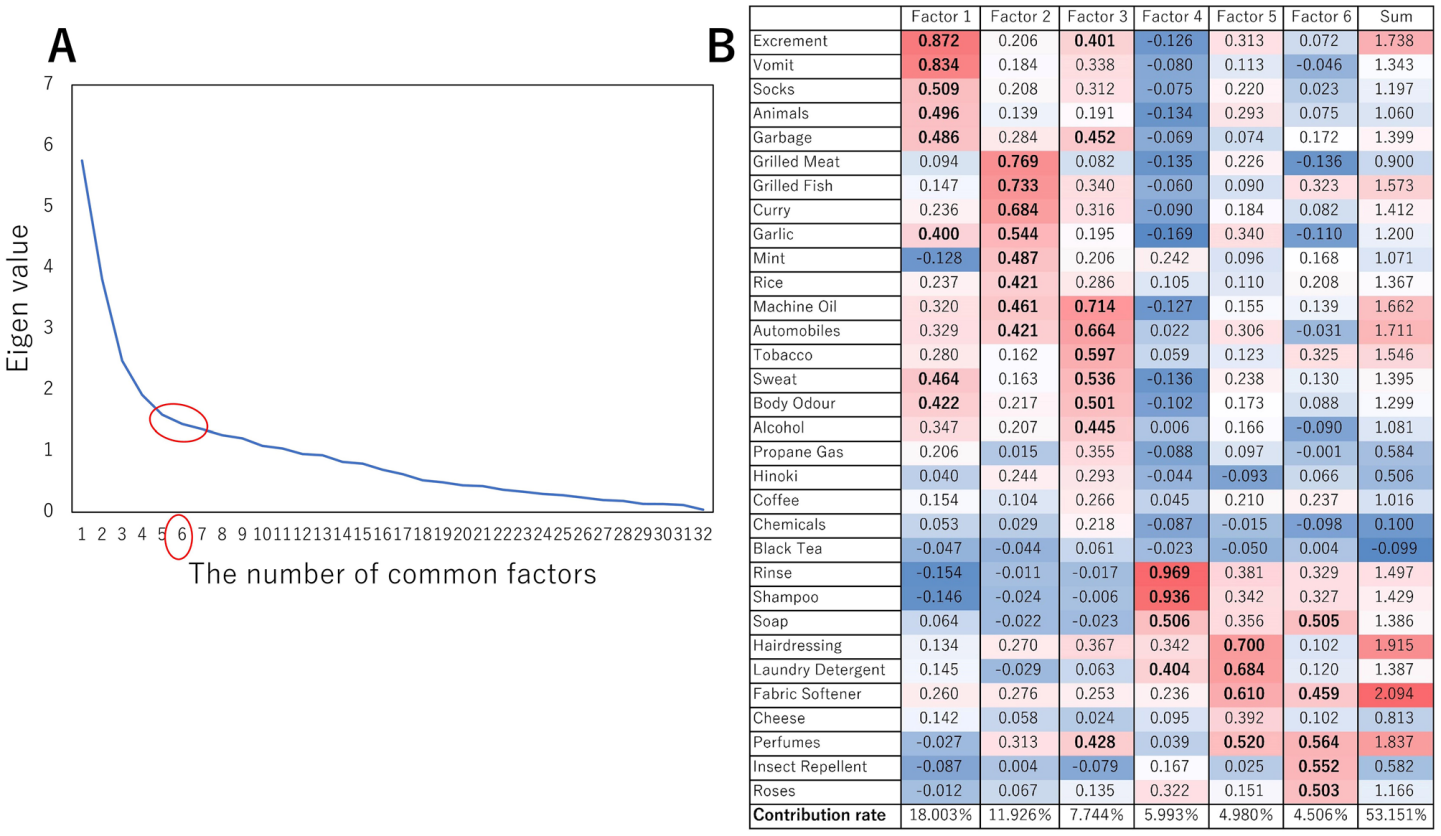
Elbow chart and factor structure matrix. (**A**) Six common factors estimated through factor analysis using the elbow chart. (**B**) Factor structure matrix and factor loading values of the six factors. Factor loading values > 0.400 are in bold. The correlations between the factors were all < 0.388. Factor 1, fetid odor; factor 2, cooking products; factor 3, oil derivatives and others; factor 4, shampoo and conditioner; factor 5, cleaning products; factor 6, perfumes, insecticides, and rose.

Factor analysis was performed on 35 items of odors, excluding three items that were not associated with migraine attacks. The analysis estimated six common factors with reference to the elbow chart (Fig. 1A).

The cumulative variance was 53.2%. The factor structure matrix and loading values on the six factors are presented in Fig. 1B. The correlation between the factors was < 0.388. Based on previous reports^9,11,12^, we defined factor 1 as fetid odor, factor 2 as cooking products, factor 3 as oil derivatives and others, factor 4 as shampoo and conditioner, factor 5 as cleaning products, and factor 6 as perfumes, insecticides, and rose.

The Spearman correlation coefficient test revealed that factors 3 (r = 0.690, P < 0.001), 1 (r = 0.472, P = 0.006), and 2 (r = 0.397, P = 0.025) seemed to be particularly important as hidden factors in headache-inducing odors, in that order. CM was likely induced by factor 5 compared with EM (P = 0.037). Factors 2, 3, 4, 5, and 6 had a stronger influence on headache aggravation (P < 0.001, P < 0.001, P = 0.002, P < 0.001, and P < 0.001, respectively).

### Clustering analysis

**Figure 2:**
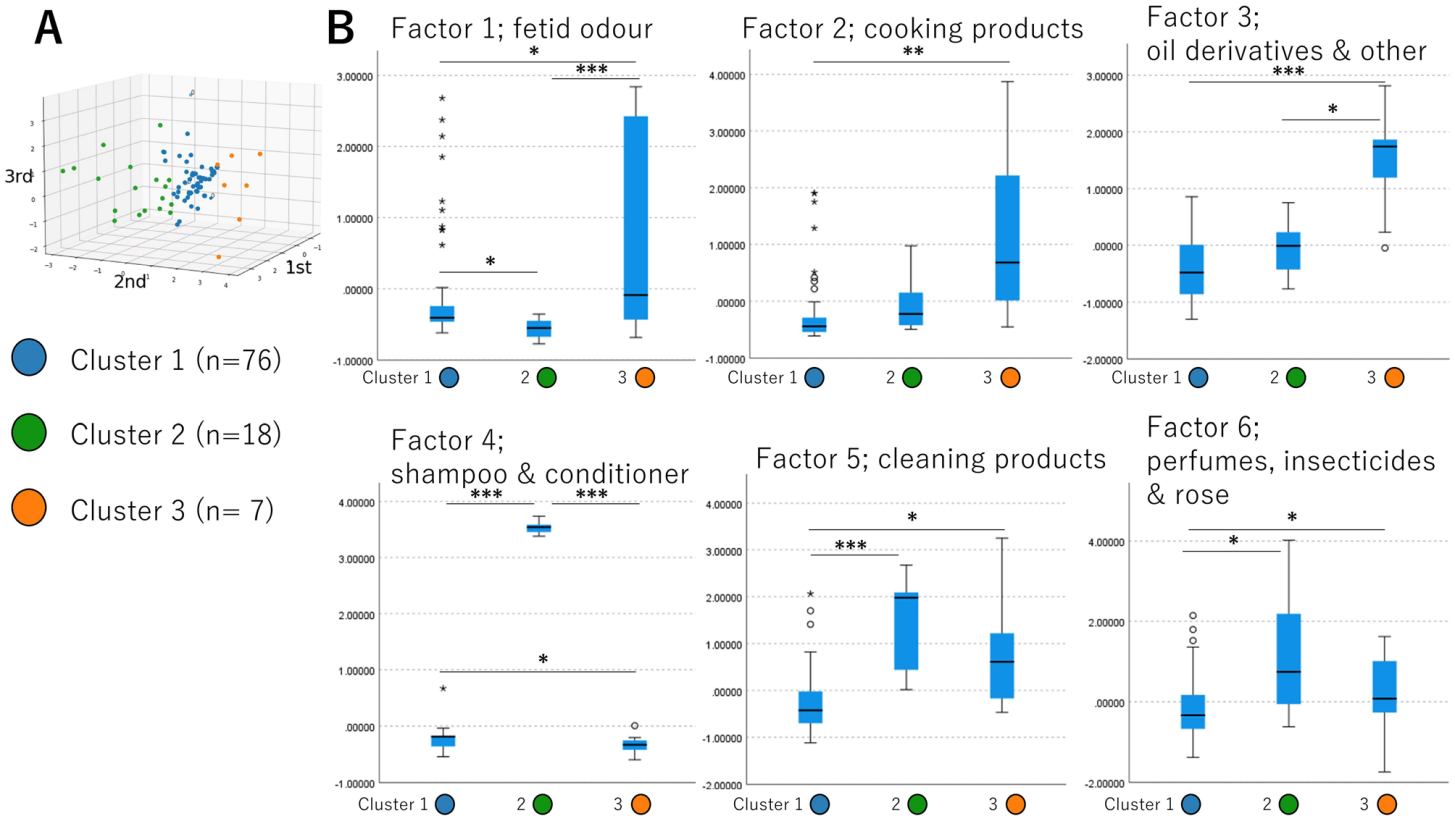
Clustering results and their barycenters. (**A**) Clusters plotted in three-dimensional space; axes were calculated using principal component analysis. Cluster 1, 76 individuals (blue); cluster 2, 18 individuals (green); cluster 3, 7 individuals (orange). (**B**) The sensitivities against the common factors were statistically analyzed. Cluster 1 had a slightly stronger sensitivity than cluster 2 to factor 1 and than cluster 3 to factor 4. Cluster 2 had a strong sensitivity to factors 4, 5, and 6. Cluster 3 had a strong sensitivity to factors 1, 2, 3, 5, and 6. *P < 0.050, **P < 0.010, ***P < 0.001.

The six common factors were used for k-means ++ clustering. The biggest silhouette score^21^ was 0.471268, suggesting that 3 is the appropriate number of clusters. The clusters were plotted in the three-dimensional space, with the axes calculated using principal component analysis consisting of the six factors (Fig. 2A and Supplementary Video S1). The barycenter of each cluster is presented in Table 3.

**Table 3:** Barycenter of each cluster.

	Cluster 1 (blue) (n = 76)	Cluster 2 (green) (n = 18)	Cluster 3 (orange) (n = 7)	P value^†^	P value^‡^
Factor 1: fetid odor	− 0.56205	− 0.85752	− 0.15133	0.002	1 > 2: P = 0.020
					3 > 2: P < 0.001
					3 > 1: P = 0.019
Factor 2: cooking products	− 0.26273	− 0.04106	1.12527	< 0.001	3 > 1: P = 0.001
Factor 3: oil derivatives and other	− 0.35991	− 0.06059	1.54317	< 0.001	3 > 2: P = 0.027
					3 > 1: P < 0.001
Factor 4: shampoo and conditioner	− 0.24726	3.53210	− 0.32960	< 0.001	3 > 2: P < 0.001
					2 > 1: P < 0.001
					3 > 1: P = 0.040
Factor 5: cleaning products	− 0.28847	1.38852	0.67802	< 0.001	2 > 1: P < 0.001
					3 > 1: P = 0.040
Factor 6: perfumes, insecticides, and rose	− 0.16231	1.20098	0.21872	0.008	2 > 1: P < 0.011
					3 > 1: P = 0.034

^†^Tested using Kruskal–Wallis test.

^‡^Tested using Mann‒Whitney *U* test after Kruskal‒Wallis test.

The clustering results are presented in Fig. 2A. Sensitivity to common factors was statistically analyzed, and the results are presented in Fig. 2B.

## Discussion

**Figure 3:**
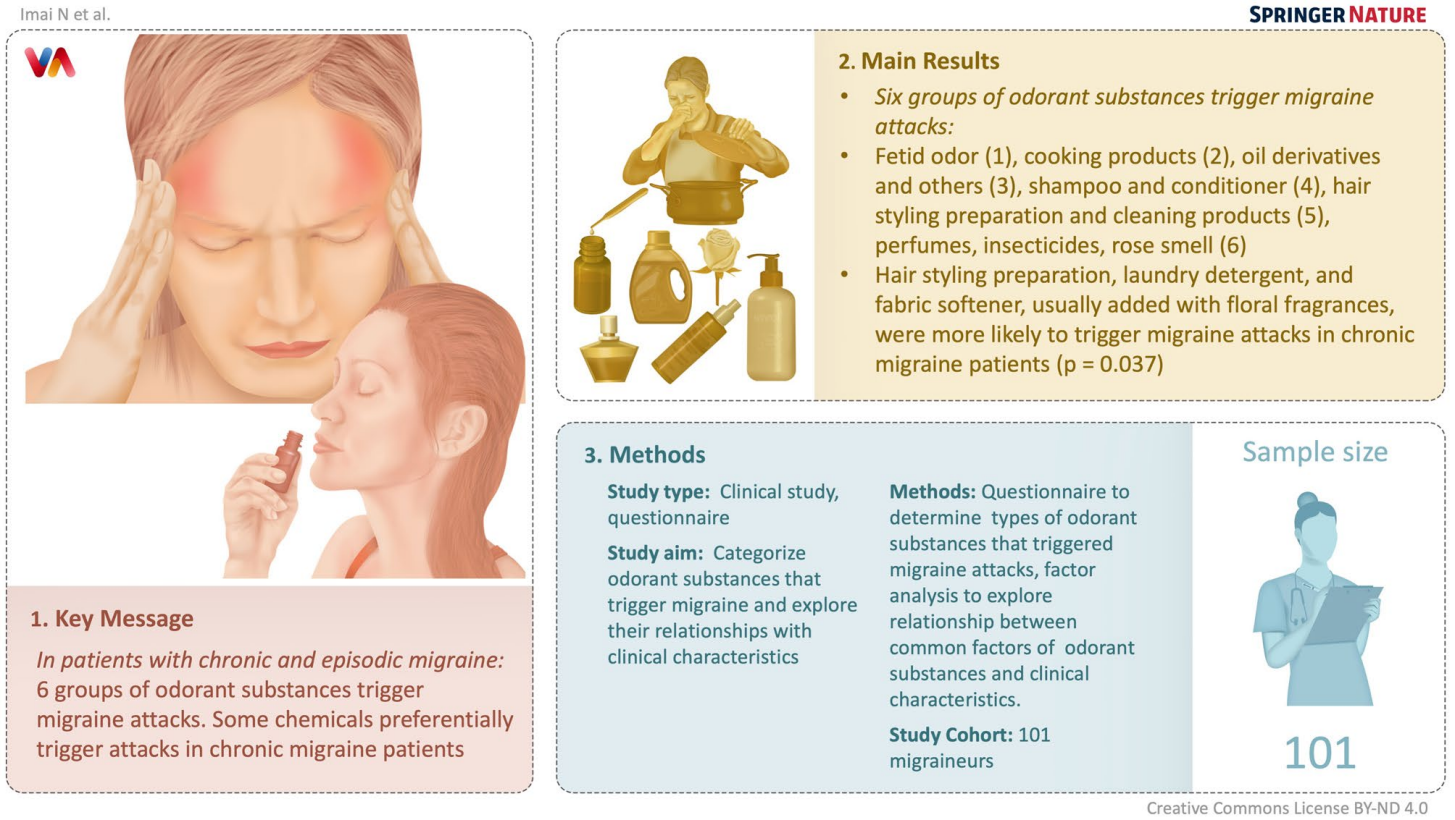
Summary of this study.

This study analyzed 101 migraineurs to identify odors associated with migraine attacks. The study used factor analysis to group the odorants into six categories, including fetid odor; cooking products; oil derivatives; shampoo and conditioner; cleaning products; and perfumes, insecticides, and rose scent. The study found that factor 5, which included floral fragrances in hair styling preparations, laundry detergent, and fabric softener, usually those with floral fragrances, was more likely to be associated with migraine attacks in patients with CM than in those with EM. Overall, the study suggests that some chemicals are more likely to be associated with migraine attacks in patients with CM (Fig. 3).

Olfaction occurs when an odorant substance binds to a receptor within the nasal cavity, transmitting a signal through the olfactory system. Odors can stimulate nasal trigeminal receptors^14,17^. Since most odors are bimodal or activate the trigeminal and olfactory nerves, there are two possible mechanisms for inducing migraine attacks. Odor threshold, discrimination, and identification abilities are lower in migraineurs with and without osmophobia than in healthy controls, and discrimination ability was especially lower in patients with osmophobia^11^.

Factor analysis was used to determine common factors in the odor associated with migraines. If the results indicate that a particular odorant substance is associated with migraine, it may be possible to prevent migraine attacks by avoiding the odorant substance. In addition, some patients are more sensitive to an odorant substance than others, and we observe this in daily clinical practice. Clustering was performed to group patients’ sensitivity to an odorant substance, which may allow us to tentatively consider the relationship between the odorant substance and the onset/severity of migraine headaches. The results of the factor analysis were included in the clustering analysis, and not all of the original odorant substance variables were included in the calculation. This was done to avoid the disadvantages of dimensions such as distance concentration and data sparsity due to the large number of variables. Our factor analysis showed that odor-associated migraine attacks could be estimated among the six clusters. Each cluster may involve different olfactory, trigeminal, and pheromone system rates.

The odors of factors 4 and 5 are predominantly flora-based and overlapping. The reason they were split into different groups in the factor analysis may have to do with the different situations in which they smelled the odorant. Factor 4 is used in the bath or shower, whereas factor 5 is used in the general environment. The possibility that differences in environmental conditions (such as temperature and humidity) and mental state, i.e., people are generally more relaxed while bathing, were mathematically calculated separately was considered.

Compared with EM, CM was likely to be associated with factor 5. Factor 5 included hairdressing products, laundry detergent, and fabric softener; thus, factor 5 was defined as cleaning products. Odors in cleaning products arise due to the use of odorant substances such as floral fragrances (rose oxide)^22^. Chronification of migraine may increase sensitivity to certain chemicals.

Non-parametric analysis showed that body odor or garbage was associated with migraine attacks in women only. Savic et al. reported the sex-specific effects of pheromones^18^. The hypothalamus is activated in women and men by the smell of an androgen-like and estrogen-like substance, respectively. Body odor is mainly induced by axillary odor, foot odor, and diacetyl decomposed by lactic acid, occurring in the occiput and around the neck^23^. Axillary odor is caused by a combination of sweat gland secretions. The exact composition of human sweat contains a mixture of different compounds, including androgen-like androstenone, androstanol, and androstadienone^24,25^. The reason why body odor was associated with migraine attacks in women only is presumed to be related to the sex-specific effects of pheromones. The main component of garbage is methyl mercaptan, which is not a pheromone^23^. In Japan, women usually take out the garbage and have more contact with it. This social context could be the reason that garbage was associated with migraine attacks only in women.

The non-parametric analysis also showed that the patients with migraine attacks associated with tobacco or soap were significantly younger than those with attacks associated with other factors. The chemical substances in tobacco are different from those in soap. Tobacco smoke contains more than 4,000 chemicals, mainly nicotine, tar, carbon monoxide, carbon dioxide, nitrogen oxides, ammonia, hydrogen sulfide, aldehydes, and ketones^23^. The scent of soap is derived from synthetic fragrances such as floral and aldehyde bouquets^24^. Therefore, it is unlikely that the chemical substances common to both tobacco and soap were associated with more migraine attacks among younger migraineurs than older migraineurs. We hypothesize that younger people have less exposure to tobacco and soap odors and are, therefore, more sensitive to unfamiliar odors. This can be due to various reasons. First, there is lower exposure to tobacco odor in public places than that in the past, and smoking rates are declining gradually^26^. Second, the use of solid soap is higher among older people, whereas younger people often use liquid body soaps, which vary from floral to citrus, herbal, and fruit scents. The strength of the odor also varies (from unscented and slightly scented to scented)^27,28^.

This study has limitations. It was conducted at a single headache clinic with the role of a regional headache center. This setting limits the generalizability of the results and may introduce a selection bias toward patients with more severe outcomes. The main limitations were the retrospective design of the study and the use of a questionnaire to retrospectively identify potential associations in their cohort. This limitation could lead to misattribution or recall bias. Osmophobia is reported in a large number of patients, and early manifestation of osmophobia may be the reason for a large proportion of self-reported odorants as triggers, i.e., an odorant with an associations vs. early manifestation of osmophobia^29,30^. Prospective confirmation is needed to determine whether or not this is an actual association.

In conclusion, this retrospective study suggests that odors associated with migraine attacks could be categorized into six groups using factor analysis. These results suggest that the chronification of migraine is associated with increased sensitivity to certain chemicals. Further prospective studies are needed to confirm these findings.

## Methods

### Patients

Patients with migraine who consulted the Department of Neurology, Japanese Red Cross Shizuoka Hospital, were enrolled from April 28 to September 8, 2020. All patients fulfilled the criteria of the International Classification of Headache Disorders, 3rd Edition for migraine without aura, migraine with aura, or CM^31^, as determined by a headache specialist certified by the International Headache Society. The exclusion criteria were as follows: tension-type headache for > 5 days per month, history of any other primary headache, pregnancy, breastfeeding, cardiovascular or cerebrovascular disease, uncontrolled psychiatric disorder, and drug abuse. The Japanese Red Cross Shizuoka Hospital Ethics Committee approved the study protocol (approval number 2021-05), and the study was conducted in accordance with the Helsinki II Declaration of 1964 with later revisions. Written informed consent was obtained from all participants. The sample size was set at 100 cases based on other studies of odor^9,10^.

### Questionnaire

A questionnaire was used to determine whether odors induced migraine attacks, which odors or location induced migraine attacks, and how the participants cope with the odors. The questionnaire included 35 items of odors selected according to previous studies to determine the types of odors that trigger migraine attacks^11,12,14,15^. The locations were homes, workplaces, restaurants, and hospitals. The questionnaire items for preventing odors were masks, air cleaners, and deodorant spray. Free-text fields were created for all items so that participants could describe odors, locations, and remedies not included in the items.

### Statistical analyses of demographics and relationships between clinical characters and odors

Demographics and relationships between clinical characters and odors were analyzed using t-tests for continuous data with normal distribution, Mann‒Whitney U test or Kruskal‒Wallis test for non-parametric variables, and Fisher’s exact test for classified data using the SPSS Statistics 20.0 (IBM Corp., Armonk, NY, USA). The significance level was defined as P < 0.05. We classified migraine without aura and migraine with aura as EM and compared them with CM.

We conducted a factor analysis using the maximum-likelihood method with the promax rotation using SPSS Statistics 28.0 (IBM Corp.) to explore common factors of odors that cause migraine. The number of common factors was decided with reference to the elbow chart. The relationship between the common factors and migraine occurrences was tested using Spearman’s correlation coefficient. Furthermore, to investigate the migraineurs’ sensitivities against the common factors calculated by the factor analysis, we performed non-stratified clustering using k-means ++ using Python 3.9.0, scikit-learn 0.24.1, and Matplotlib 3.4.3. The clusters were plotted in the three-dimensional space, the axes of which were calculated using principal component analysis^32,33^.

## Supplementary Information

Supplementary Legends.

Supplementary Video 1.

## Data Availability

All data generated or analyzed during this study are included in this published article [and its Supplementary Information files].
