# Supplementary material for: Classification of odors associated with migraine attacks: a cross-sectional study
Source: Sci Rep. 2023 May 25;13:8469. doi: 10.1038/s41598-023-35211-7 (PMC10213061; doi:10.1038/s41598-023-35211-7)
Supplement: Supplementary file 1 — Supplementary Legends. [file 41598_2023_35211_MOESM1_ESM.docx]

**Supplementary Video 1**

**Clustering results**

The clusters are plotted in the three-dimensional space, of which the axes were calculated by the principal component analysis. Cluster 1 has 76 individuals (blue), cluster 2 has 18 individuals (green), and cluster 3 has 7 individuals (orange).
